# Unraveling ultrasonic assisted aqueous-phase one-step synthesis of porous PtPdCu nanodendrites for methanol oxidation with a CO-poisoning tolerance

**DOI:** 10.1016/j.ultsonch.2023.106494

**Published:** 2023-06-17

**Authors:** Qingqing Lu, Xilei Gu, Jiaojiao Li, Wenpeng Li, Rafael Luque, Kamel Eid

**Affiliations:** a Engineering & Technology Center of Electrochemistry, School of Chemistry and Chemical Engineering, Qilu University of Technology (Shandong Academy of Sciences), Jinan, China; b Peoples Friendship University of Russia (RUDN University), 6 Miklukho Maklaya str., 117198 Moscow, Russian Federation; c Universidad ECOTEC, Km 13.5 Samborondón, Samborondón EC092302, Ecuador; d Gas Processing Center (GPC), College of Engineering, Qatar University, Doha 2713, Qatar

**Keywords:** Porous Pt alloy, Methanol oxidation reaction, Fuel cell, PtPdCu nanodendrites, Electrocatalyst, CO-poisoning tolerance

## Abstract

The tailored design of tri-metallic Pt-based porous nanodendrites (PNDs) is crucial for green energy production technologies, ascribed to their fancy features, great surface areas, accessible active sites, and stability against aggregation. However, their aqueous-phase one-step synthesis at room temperature remains a daunting challenge. Herein, we present a facile, green, and template-free approach for the one-step synthesis of PtPdCu PNDs by ultrasonication of an aqueous solution of metal salts and Pluronic F127 at 25 ℃, based on natural isolation among nucleation and growth step driven by the disparate reduction kinetics of the metals and acoustic cavitation mechanism of ultrasonic waves. The resultant PtPdCu PNDs formed in a spatial nanodendritic shape with a dense array of branches, open corners, interconnected pores, high surface area (46.9 m^2^/g), and high Cu content (21 %). The methanol oxidation reaction (MOR) mass activity of PtPdCu PNDs (3.66 mA/µg_Pt_) is 1.45, 2.73, and 2.83 times higher than those of PtPd PNDs, PtCu PNDs, and commercial Pt/C, respectively based on equivalent Pt mass, which is superior to previous PtPdCu catalysts reported elsewhere, besides a superior durability and CO-poisoning tolerance. This study may pave the way for the controlled fabrication of ternary Pt-based PNDs for various electrocatalytic applications.

## Highlights

• Sonication-assisted green, and one-step synthesis of PdPtCu PNDs in 20 min at 25 °C.
• Various experiments were done to confirm the formation mechanism of PtPdCu PNDs.
• The MOR activity of PtPdCu PNDs was higher than PtPd PNDs, PtCu PNDs, and Pt/C.
• PtPdCu PNDs outperformed all previously reported PtPdCu nanostructures for MOR.
• PtPdCu PNDs remain durable, maintained shape and composition after MOR durability.

## Graphical abstract

**Figure:**
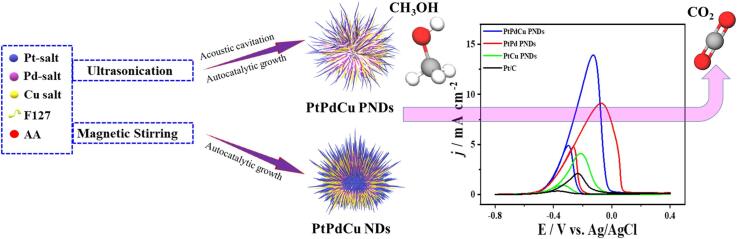

## 1 Introduction

The inevitable usage of non-renewable fossil fuels leads to their depletion, huge greenhouse gas emissions, and subsequent environmental issues [1], [2], [3], [4], [5], raising the need for green energy resources. The methanol-based fuel cell (MFC) is an auspicious green energy production [1], [2], owing to its outstanding energy yield, earth-abundance, low cost, and low toxicity, besides ease of preparation, handling, and storage [3], [4], [5]. However, MFC remains impractical, due to the exorbitant cost, earth scarcity, self-poisoning, and ceaseless price rise of Pt, which is the main catalyst for methanol oxidation reaction (MOR) [1]. Tailoring morphologies (i.e., porosity, dimensions, and surface feature) and alloying Pt with one or two lower-cost and earth-abundant metals (i.e., Cu, Sn, Mo, Ni, and Fe) with or without support are the conceivable solutions to conquest these fences and improve the catalytic activity towards MOR and other catalytic applications [6], [7], [8], [9]. Ubiquitously, the formation of porous ternary Pt-based alloy alters the d-band center of Pt, resulting in easing adsorption of reactants and desorption of poisoning intermediates during the MOR [10], [11], [12]. Distinct from binary Pt-based catalysts, the ternary system has many advantages that facilitate C-H bond cleavage in methanol oxidation under a lower applied potential and superior tolerance of CO-like intermediates' poisoning [13], [14], [15]. Also, porous ternary Pt alloy endows tunable and facile adsorption and activation of reactants to promotes the dissociation of the water molecule to generate active OH* species needed for accelerating the MOR kinetics along with easing desorption of the poisoning and intermediate species. Meanwhile, the strong bond between Pt and the other two metals stabilizes it against chemical oxidation or etching in electrolyte along with the balance between the quick MOR process and oxidative removal of intermediates, so the MOR activity and durability of porous ternary Pt system are always superior to binary Pt-based catalysts [16], [17], [18]. The specific activity of Pt_0.68_Cu_0.18_Ru_0.14_ NFs is 7.65 mA/cm^2^, which was 2.7, 4.1, and 6.0 times greater than those of Pt_0.79_Cu_0.21_ NPs, PtRu/C, and commercial Pt/C, respectively, besides higher durability and reduced CO-poisoning [19]. The MOR mass activity (MA) of Pt_1_Cu_0.84_Ni_0.11_ nanowires (2.10 A/mg_Pt_) was higher than those of Pt_1_Cu_0.83_ nanowires, Pt_1_Ni_0.28_ nanowires, Pt nanowires, and Pt/C catalyst by 1.94, 2.44, 3.6, 4.37 times, respectively, owing to the multimetallic electronic effect which strengthened the binding of *OH, but weakened the binding of *CO, resulted in accelerating the MOR kinetics [20].

Ternary PtPdCu alloy is among the most promising electrocatalysts for MOR, resulting from its great synergism and multifunctional mechanism [21], [22], [23], [24]. Particularly, the alloying effect of Pt-Pd enhances the adsorption of reactants; meanwhile, Pt-Cu generates the oxygenated species (i.e., O_2_ and OH) that allows cleavage of C-H bond on its adjacent Pt sites and consequently accelerates the MOR kinetics at a low potential. Notably, porous ternary PtPdCu catalysts are rarely reported for catalytic applications (∼42 articles, including only 15 articles for MOR) according to the web of sciences as far as we found. Ternary Pt_34_Pd_33_Cu_33_ nanocrystals [25], PtPdCu/rGO [26], Pt_5_PdCu_5_ spherical-network [27], mesoporous PtPdCu spheres [28], nanoporous PtPdCu spheres [29], and PtPdCu/TiN nanorods were prepared by sputter-deposited on TiN exbihited higher MOR activity than their counterparts binary and Pt/C catalysts [30]. For example, The MOR MA of spatial PtPdCu porous nanowires (0.15 mA/µg_Pt_) formed by the chemical reduction in Triton X-114 was 6.5 times higher than Pt/C [31]. Also, PtPdCu hollow sponges formed by the template-based method and HNO_3_ etching for 2 days had increased MA (1.34 mA/μg_Pt_) by 2.2, and 3.9 times than those of PtCu-HS and Pt/C, respectively [32].

Ternary porous branched structures, especially porous nanodendrites (PNDs), encompass myriad of advantages, such as the great surface areas, rich edges, and interior/exterior cavities, which adsorb methanol and ease their diffusion, thus maximize the usage of metals with accelerating mass transfer during the MOR [33], [34], [35], [36]. For instance, the MOR MA of Pt_5_PdCu_5_ hexapods (0.97 mA/μg_Pt_) obtained by the solvothermal method at 200 ℃ for 3 h was 5.4-fold higher than that of Pt/C (0.18 mA/μg_Pt_), due to the ligand effect and hexapod structure [37]. The MOR MA of PtPdCu NDs (0.52 mA/μg_Pt_) formed by the chemical reduction and etching at 90 °C for 2.5 h was 1.52 times of PtPdCu nanocubes (0.341 mA/μg_Pt_) and 2.97 times of Pt/C [38]. PtPdCu concave nanooctahedra formed by the seed-mediated growth at 140 °C for 4 h revealed MOR MA of 529.5 A/g_Pt,_ which was 1.29 and 4.13 times higher than PtCu NDs and Pt black, respectively [39]. PtPdCu NDs formed by the template-based and electrodeposition method had enhanced MOR MA (688 mA/mg_Pt_), which was 1.78-folds and stability by 5.02-folds of Pt/C [40]. PtPdCu hexapod concave rhombic dodecahedrons formed by the autoclave at 180 °C for 24 h showed MOR MA of 2.23 mA/µg_Pt_, which outperformed Pt/C by 4.1 folds [41]. Hexameric octahedral Pt_0.75_Pd_0.13_Cu_0.12_ formed by seed-mediated growth in octadecylamine and cyclohexane at 270 °C showed a current density of 3.47 mA/cm^2^, which was 3.8 times higher than Pt/C catalysts [42]. The MOR MA of PtPdCu NDs (1.447 mA/μg_Pt_) obtained by the chemical reduction at 95 °C for 40 min was compared with those of PtPd NDs (1.066 mA/μg_Pt_), PtCu NDs (0.809 mA/μg_Pt_), PdCu NDs (0.626 mA/μg_Pt_), and Pt black (0.568 mA/μg_Pt_) [43]. These approaches entail multiple reaction steps, heating, and organic solvents to form PtPdCu nanocrystals for the MOR. Unlike previous reports, the sonochemical method with its acoustic cavitation effect can allow the simple and fast aqueous-phase one-step synthesis of ternary PNDs, but it is rarely reported [44]. Mainly, the reduction kinetics under sonication is substantially higher than that without sonication and can allow reduction and alloying of Pt with transition metals even using a weak reducing agent because the oscillation frequency of ultrasound creates massive bubbles, which undertakes oscillatory growth and go through a rapid inertial overgrowth and finally collapse after reaching a critical size (tens of micrometers) [44], [45], [46], [47], [48]. This collapsing discharges the concentrated energy stored in the bubbles promptly at a spontaneous heating/cooling rate (∼≥ 10^9^ K/s) and the generation of a localized micro-scale with great energy (∼5000 K and ∼1000 bar) [49], [50]. In the system, sonochemical method involves the dissociation of H_2_O to generate reactive species (i.e., H^⋅^ and OH^⋅^), which can form (H_2_ and/or H_2_O_2_) or HO_2_^⋅^ via coupling H^⋅^ with dissolved O_2_, that serves as strong oxidants or reductants under sonication to accelerate the reduction process of metal precursors in aqueous solution [49], [50]. Thereby, using ultrasonication, we synthesized mesoporous PtPd PNDs [6], PtNi PNDs [51], PtCu PNDs [52], and PtPdRu PNDs [53] with ordered pores and outstanding surface area of 58–100 m^2^/g in the presence of various non-ionic copolymers, which exhibited enhanced methanol/ethanol oxidation performance significantly than Pt/C catalyst. Despite the substantial achievements in synthesizing ternary PtPdCu PNDS, their one-step synthesis with tunable composition and ordered porosity in an aqueous phase solution using sonication remains a challenge, due to the complications related to the reduction of three metals with disparate standard reduction potential and their dissimilar interaction with the structural-directing agents. Also, the effect of ultrasonic irradiation on the ternary Pt-based nanocrystals is still ambiguous and not deciphered well.

Here, we present a simple approach for the one-step production of PtPdCu PNDs with a well-defined shape and composition driven by the simple ultrasonication of an aqueous solution of Pt/Pd/Cu precursors, ascorbic acid, and F127 at room temperature. Unlike previously reported methods for PtPdCu, our approach is facile, one-step, aqueous-phase without the need for heating and organic solvents for the production of PtPdCu PNDs with a high Cu content (21 %) and dispersed pores in the entire structure (Table S1). We have conducted various experiments to understand the effect of sonochemical waves on the fabrication process of PtPdCu PNDs. The electrocatalytic activity and durability of thus obtained PtPdCu PNDs were examined relative to PtPd PNDs, PtCu PNDs, and commercial Pt/C catalyst towards MOR. Also, the CO-poisoning tolerance and structural/compositional stability after MOR tests were also studied. These merits endowed PtPdCu PNDs with superior MOR activity than all previously reported PtPdCu nanocrystals (Table S1).

## 2 Experimental and methods

### 2.1 Chemicals and materials

Potassium tetrachloroplatinate(II) (K_2_PtCl_4_, ≥ 99.99 %), sodium tetrachloropalladate(II) (Na_2_PdCl_4,_ ≥ 99.99 %), copper chloride dihydrate (CuCl_2_·2H_2_O ≥ 99 %), L-ascorbic acid ((AA), ≥ 99 %), Pluronic F127 ((C_3_H_6_O·C_2_H_4_O)_x_, *M*_wt_.12600 g/mol), and potassium hydroxide (KOH ≥ 85 %, pellets) were bought from Sigma-Aldrich Chemie GmbH (Munich, Germany).

### 2.2 Synthesis of porous PtPdCu PNDs

PtPdCu PNDs were synthesized by the ultrasonication approach based on our previous method, but with some modifications [53]. This includes the sonication of Na_2_PdCl_4_ (2.5 mL, 20 mM), CuCl_2_ (2.5 mL, 20 mM), K_2_PtCl_4_ (4 mL, 20 mM), F127 (0.1 g), and AA (1.0 mL, 0.4 M) for 20 min at 25 °C. Consequently, the PtPdCu PNDs were purified by centrifugation at 10,000 rpm and washing with double-deionized H_2_O. The sonication was conducted in (Ultrasonic cleaner bath 1.9 L, Fisher Scientific FS60H, USA) at Frequency 80 kHz and voltage amplitude of 5.0 mV.

### 2.3 Synthesis of PtPd PNDs

PtPd PNDs were formed by the sonication of NaPdCl_4_ (3.5 mL, 20 mM), K_2_PtCl_4_ (5.5 mL, 20 mM), F127 (0.1 g), and AA (1 mL, 0.4 M) in water at 25 °C for 20 min and then centrifugation and washing [6].

### 2.4 Synthesis of PtCu PNDs

PtCu PNDs were formed by the sonication of K_2_PtCl_4_ (5.5 mL, 20 mM), CuCl_2_ (3.5 mL, 20 mM), F127 (0.1 g), and AA (1 mL, 0.4 M) in water at 25 °C for 20 min and then centrifugation and washing [52].

### 2.5 Synthesis of PtPdCu NDs

PtPdCu NDs were prepared by mixing Na_2_PdCl_4_ (2.5 mL, 20 mM), CuCl_2_ (2.5 mL, 20 mM), K_2_PtCl_4_ (4 mL, 20 mM), and F127 (0.1 g) in water under magnetic stirring followed by quick addition of AA (1.0 mL, 0.4 M) for 4 h at 25°C.

### 2.6 Materials characterization

A transmission electron microscope (TEM) (TecnaiG220, FEI, Hillsboro) supplied with high-angle annular dark-field scanning TEM (HAADF-STEM) and energy dispersive spectrometer (EDS) was used. The X-ray photoelectron spectroscopy (XPS) was analyzed on a Kratos Axis (Ultra DLD XPS Kratos). The X-ray diffraction patterns (XRD) were conducted on an X-ray diffractometer (X'Pert-Pro MPD, PANalytical Co., Almelo). N_2_-physisorption isotherms were conducted on a Quantachrome Autosorb 3.01 instrument. The surface area was calculated from isotherms curves using Brunauer-Emmett-Teller (BET), and porosity was estimated by Barrett, Joyner, and Halend (BJH) method. Inductively coupled plasma-optical emission spectrometry (ICP-OES) measurements were performed on Thermo Scientific X Series 2, USA.

### 2.7 Methanol oxidation reaction

The MOR was conducted on a CHI 760E potentiostat using a three-electrode glass cell in KOH medium (See supporting information for more details).

## 3 Results and discussion

### 3.1 Characterization of nanocatalysts

**Fig. 1:**
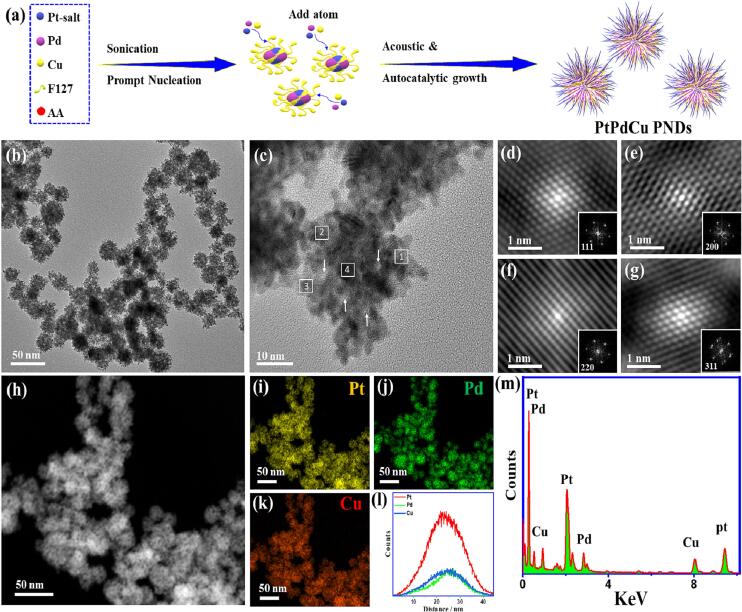
(a) Formation process, (b)TEM image, (c) HRTEM, (d-g) Fourier filtered HRTEM images of 1,2,3, and 4 areas in (c), respectively, (h) HAADF-STEM image, (i-k) element mapping analyses, (l) EDS-scan line profile, and (m) EDS of PtPdCu PNDs. The insets in (d-g) are the corresponding FFT patterns.

PtPdCu PNDs were prepared via the ultrasonic irradiation of Pt/Pd/Cu precursors in an aqueous solution comprised of F127 as a morphology-directing agent with the aid of AA as a reductant (Fig. 1a) [53]. This is driven by the spontaneous isolation of nucleation from the growth step barreled by the acoustic cavitation mechanism. The TEM image of PtPdCu PNDs showed the high-yield formation of uniform nanodendrites comprising multiple arms and inner/outer pores (Fig. 1b). The size of thus obtained PtPdCu PNDs was about 35 ± 3 nm (Fig. S1a). The high resolution TEM (HRTEM) image of an individual particle displayed its porous dendritic structure composed of 3D gathered arms with a diameter of 3 ± 1 nm (Fig. 1c). PtPdCu PNDs entail abundant interior and exterior pores with a size of 4 ± 1 nm (arrows in Fig. 1c). The resolved lattice fringes are uniform without any crystal defects or undesired microscopic phases, implying the uniformity of thus obtained PtPdCu PNDs alloys. The lattice fringes are distributed in different directions from the core to the outer shell, indicating the non-epitaxial growth of PtPdCu PNDs [53]. This results from the different reduction potentials of Pt^2+^/Pt 0.755 V versus standard hydrogen electrode (vs. SHE), Pd^2+^/Pd 0.591 V (vs*.* SHE), and Cu^2+^/Cu 0.341 V (vs*.* SHE), which lead to different reduction kinetics and subsequent growth rate.

This means that upon chemical reduction by ascorbic acid under sonication, Pt precursors will be preferentially reduced first to form Pt nuclei that serve as seeds and provide favorable sites for consecutive atomic addition of Pd and Cu. So, the growth will be non-epitaxial, and the lattice fringes will be in different directions. The Fourier filtered (FFT) lattice fringes images demonstrated the most exposed facets are {1 1 1}, {2 2 2}, {3 1 1}, {2 0 0} of face-centered-cubic (*fcc*) Pt, implying the single crystallinity of PtPdCu PNDs (Figs. 1d-1g), demonstrating the formation of PtPdCu alloys via homogenous nucleation and subsequent growth rather than random agglomeration [53]. This is further seen in the selected area electron diffraction (SAED) patterns of PtPdCu PNDs, which displays the typical rings of bright spots assigned to *fcc* Pt (Fig. S1b). This means the polycrystallinity of PtPdCu PNDs is attributed to the non-epitaxial growth, dissimilar atomic radius, electronic structure, and reduction kinetics of the three metals, as usually observed in ternary Pt-based alloys [38], [39], [40], [41], [42], [43], [53].

The HAADF-STEM analysis also reveals the formation of porous three-dimensional nanodendrites morphology with various cavities as inferred in the intense contrast among subunits distributed through the entire PtPdCu PNDs (Fig. 1h). The mapping analysis of PtPdCu PNDs indicates the existence of Pt, Pd, and Cu (Figs. 1i-1k). The EDS scanning line profile infers that PtPdCu PNDs are composed of Pt, Pd, and Cu in the form of alloy with Pt enriched shell and Pd/Cu are concentrated in the core area (Fig. 1l). The average atomic ratios of Pt/Pd/Cu in PtPdCu PNDs are about 51/28/21 at.%, respectively (Table S2) in concurrent with the EDS (Fig. 1m). The ICP-OES displays the atomic ratios of Pt/Pd/Cu in PtPdCu PNDs as 52/29/19 at.%, respectively, which is almost close to that obtained from the EDS (Table S2). The TEM images of PtPd PNDs and PtCu PNDs also display the formation of PNDs with an average diameter of 19 ± 2 and 25 ± 3 nm, respectively (Fig. S2). Notably, PtPd PNDs were more porous and branched than PtCu PNDs, plausibly aroused from the higher lattice matching of Pt with Pd than Cu.

### 3.2 Crystal structure

**Fig. 2:**
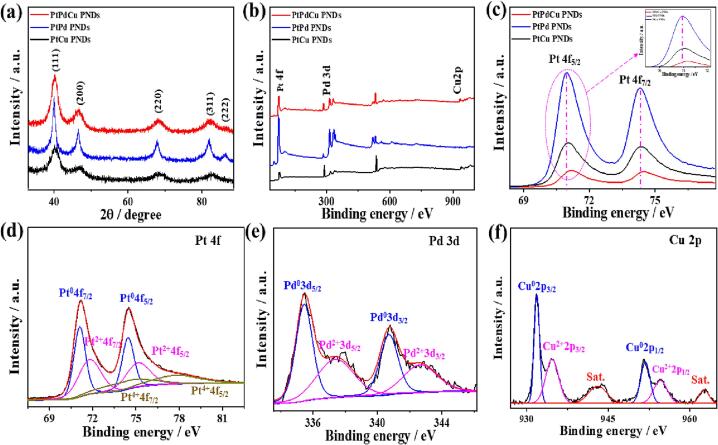
(a) Wide-angle XRD patterns, (b) XPS surveys, and (c) Pt 4f XPS spectra of PtPdCu PNDs, PtPd PNDs, and PtCu PNDs. XPS spectra of (d) Pt 4f, (e) Pd 3d, and (f) Cu 2p of the PtPdCu PNDs.

The XRD patterns of PtPdCu PNDs, PtCu PNDs, and PtPd PNDs showed the peaks of *fcc* Pt with dominant {1 1 1} facet, inferring the formation of single-crystalline alloy phase (Fig. 2a) [53]. This is seen in the lack of phases for mono Pt, Pd, Cu, or their oxides, indicating possible mixing of the Pt/Pd/Cu at the atomic level instead of segregation [6], [52], [53]. Notably, the diffraction patterns of PtPdCu PNDs slightly shifted positively to lie between pure PtCu (ICDD: 04-0802) and PtPd PNDs facets (ICDD: 46-1043). This conceivably originated from the integration of Pd and Cu into Pt lattice structures, leading to a slight increment in the Pt-Pt interatomic distance and subsequent lattice contraction [6], [52], [53]. That is evidenced in the lower lattice parameter value (*a*) of PtPdCu PNDs (0.37 nm) than that of pure PtCu (0.39 nm) and PtPd PNDs (0.38 nm) as usually observed in multimetallic Pt-based nanostructures [37], [38], [39], [40], [41].

### 3.3 Electronic structure and valence state

The XPS analysis revealed the coexistence of (Pt 4f, Pd 3d, and Cu 2p) core-level in PtPdCu PNDs, (Pt 4f and Pd 3d) in PtPd PNDs, and (Pt 4f and Cu 2p) in PtCu PNDs, inferring the formation of multimetallic PNDs alloys (Fig. 2b). The surface compositions of PtPdCu, PtPd, and PtCu PNDs are determined by the XPS (Table S2). Notably, the Pt 4f of PdPtCu were slightly shifted to higher binding energy relative to Pt 4f of PtPd PNDs and PtCu PNDs, due to the electronic interaction and charge transfer between ternary Pt, Pd, and Cu elements (Fig. 2c) [6], [52], [53]. The red shift in the binding energy indicates down-shift in the d-band center of Pt, which is highly beneficial for tunable adsorption of reactants alongside high intermediates tolerance over PtPdCu PNDs surface during MOR [10]. Thereby, as the d-band center of PtPdCu PNDs downshifted more relative to PtPd PNDs and PtCu PNDs, it is expected to show superior MOR activity. Owing to the higher electron transition between Pt and Cu, the binding energy of Pt 4f in PtCu is shifted positively than that in PtPd PNDs.

The Pt 4f spectra of PtPdCu PNDs reveal mainly (Pt 4f_7/2_ and Pt 4f_5/2_) assigned to Pt^0^ as the main metallic phase alongside with minor oxide phases of Pt^2+^ and Pt^4+^ (Fig. 2d). The same phases were obtained in PtPd PNDs and PtCu PNDs (Fig. S3a and 3c). Likewise, Pd 3d spectra were fitted into (Pd3d_5/2_ and Pd3d_3/2_) of Pd^0^ as the major phase besides Pd(II) as a minor phase (Fig. 2e). The same peaks were also resolved in PtPd PNDs (Fig. S3b). The presence of Pt-O and Pd-O is attributed to the ease of oxidation in the air [31], [32], [37], [38], [40], [41], [43]. The fitting of Cu 2p spectra revealed (Cu 2p_3/2_ and Cu 2p_1/2_) of Cu^0^ as the dominant phase and Cu^2+^ as the weak phase (Fig. 2f). The same peaks of Cu 2p were recorded in PtCu PNDs (Fig. S3d). The presence of Cu^2+^ may result from the adsorbed Cu^2+^ species or the oxidation of metallic Cu on the catalyst surface, but Cu mainly exists in the metallic phase as reflected in the higher intensity of Cu^0^ peak than that of Cu^2+^. This is further supplemented by the Auger electron spectrum of Cu LM2, which showed a dominant peak at the kinetic energy of 568 eV assigned to Cu^0^ valence (Fig. S3e), implies the formation of PtPdCu alloy in line with the XRD results. The presence of Pt, Pd, and Cu in the metallic phase is aroused from the substantial reduction power of AA under the acoustic cavitation effect of sonication, resulting in the formation of pure PtPdCu alloy [6], [52], [53].

### 3.4 Porosity and surface area

**Fig. 3:**
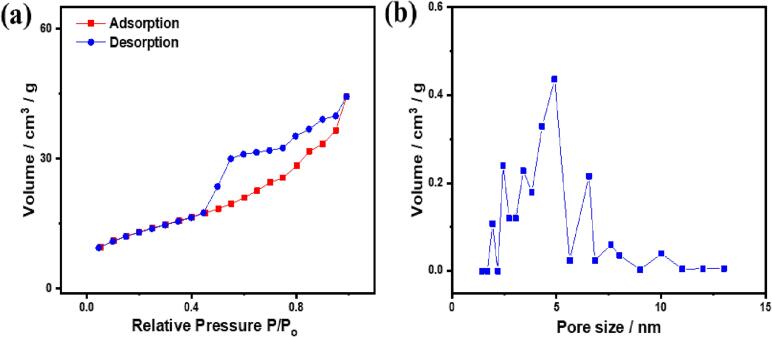
(a) N_2_-adsorption/desorption isotherm and (b) pore volume of PtPdCu PNDs.

The N_2_-adsorption/desorption isotherm of PtPdCu PNDs displays that the hysteresis loop close to the type IV curve entails two-step capillary condensation at *P*/*P*_0_ < 0.99 and *P*/*P*_0_ > 0.4, besides a prompt drop in the desorption curve at 0.52, which are the main features of bimodal pore-size distribution (Fig. 3a) [27], [52], [54].The BET surface area was about 41.2 m^2^/g and BJH pore volume of 0.04–0.48 cm^3^/g with various pore sizes in the range of 1.8 to 10 nm (Fig. 3b). The large pore volume plausibly originated from the self-assembly of F127 and the acoustic cavitation effect of the sonochemical wave [28], [52], [53]. The multiple pores are highly beneficial for accelerating guest species' diffusion and maximizing elements' utilization during MOR [39], [40], [41], [42], [43], [44], [45].

### 3.5 Formation mechanism of nanocatalysts

To sort out the formation mechanism of thus formed PtPdCu PNDs, mono Pt, Cu, binary PtPd, and PtCu nanocrystals were synthesized. Pt PNDs, PtPd PNDs, and PtCu PNDs were formed with a size of ∼17 nm (Fig. S4a), ∼19 nm (Fig. S2a), and ∼25 nm (Fig. S2b), respectively. Notably, Cu nanocrystals could not form even after extending the time to 24 h, owing to the low reduction power of AA to reduce Cu salt. However, the presence of Pt nuclei or Pd nuclei can assist the reduction of Cu via the autocatalytic effect. The reduction of Pd alone formed spherical-like nanocrystals (Fig. S4b). Meanwhile, PtPdCu PNDs with a dense core and less porosity were formed using polyvinylpyrrolidone as a template instead of F127 (Fig. S4c) due to the adsorption of PVP over Pt via carbonyl group or tertiary amine group that assembled to form spatial PNDs as we explained in our previous works [6], [52], [53]. Highly agglomerated PtPdCu nanocrystals were obtained in the absence of F127, which indicates the substantial role of F127 as a template (Fig. S4d). Chain-like nanostructures were formed via the addition of HCl (0.1 M) to the reactants (i.e., F127, metal salts, and AA) solution during sonication (Fig. S4e). This is due to the sluggish reduction power under acidic conditions, which allows the oriented attachment growth mechanism. Without sonication and magnetic stirring, the resultant PtPdCu flower-like (PtPdCu NDs) with less porosity are formed, but with less porosity and branches (Fig. S4f). In addition, it needed more than 20 min to find color change and 4 h to complete reduction; meanwhile, the Cu content was only 4 %. The reduction kinetics and subsequent growth of PNDs could be revealed in the color change of the solution from yellow to brown and black within the reaction time. Notably, the reduction kinetics was in the order of PdPtCu > PtCu > PtPd, implying the significant role of sonication in accelerating the reduction kinetics.

**Fig. 4:**
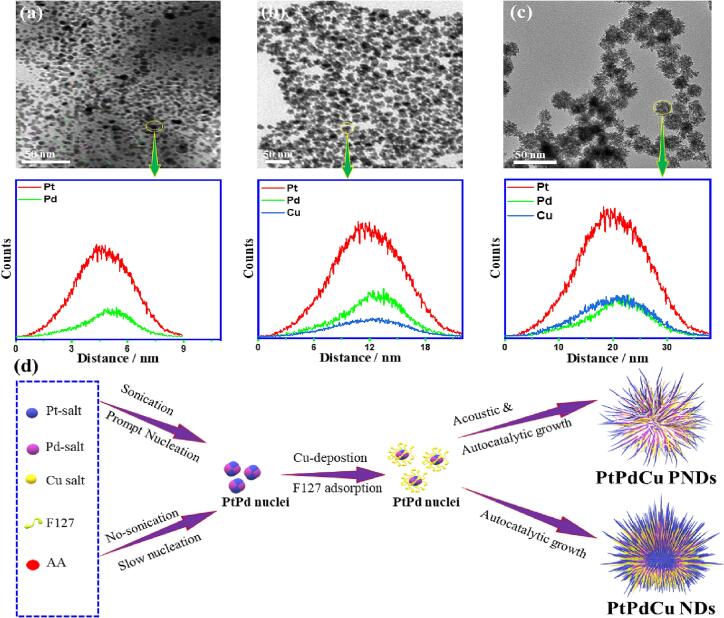
(a) TEM images of PtPdCu PNDs at 1, 5, and 10 min (a-c) and their EDS-line scan. (d) The scheme illustrates the predicted fabrication mechanism of PtPdCu PNDs.

The TEM images were conducted for various reaction intermediates at different times (i.e., 1, 5, and 10 min) after AA addition to monitor the growth of PNDs (Fig. 4). Spherical-like nanoparticles with a mean size of ∼5 nm were formed after 1 min (Fig. 4a), which grew to form branched nanocrystals, but without pore after 5 min (Fig. 4b), and finally, three-dimensional PNDs were found with larger arms and multiple pores after 10 min (Fig. 4c). We could not find any change in the morphology after 10 min. The EDS scan line profile resolved PtPd PNDs after 1 min, due to their higher positive reduction potential of Pd than Cu, while Cu is detected after 5 min, then PtPdCu with enriched Pt surface is finally formed after 10 min (Fig. 4).

This is in line with reports elsewhere, which reported the formation of PtPd nuclei that subsequently allows the reduction of the third metal by autocatalytic effect with the assistance of AA [53], [55]. Taking these results into our consideration, we could propose the formation mechanism based on the prompt nucleation of PtPd PNDs nanocrystals that act as in situ seeds for supporting deposition of unreduced metals, besides promoting the co-reduction of Pt^2+^, Pd^2+,^ and Cu^2+^ dissolved in the reaction solution via the autocatalytic effect with the assistance of AA and ultrasonic wave (Fig. 4d) [6], [52], [53]. Meanwhile, F127 is adsorbed over the surface, thus forming PtPd PNDs nuclei via hydrophobic poly(propylene oxide) group and assembled to form a porous dendritic shape [28].

The acoustic cavitation mechanism includes the prompt creation of bubbles driven by the extensive frequency oscillation of sonication. These bubbles grow oscillatory and breakdown quickly after realization of the critical diameter, resulting in the generation of enormous ultra-high energy conditions of up to ∼5000 K and ∼500 bar, which accelerates the reduction kinetics of metal precursors and forms multiple cavities during the growth step of PtPdCu, and preclude overlapping and fusion between the adjacent branches [52]. So, the isolation of nucleation from the growth step and the acoustic cavitation mechanism are the main parameter for synthesizing PtPdCu PNDs. There are various methods for synthesizing ternary PtPdCu branched nanostructures, like seed-mediated growth, template-based, etching, and solvothermal methods, but the aqueous phase one-step synthesis at room temperature with controllable compositions and ordered porosity remains a daunting challenge [37], [38], [39], [40], [41], [43]. This is aroused from the difficulties involved in co-reducing three metal precursors with disparate reduction potential, besides their mutual complexation with the surfactants. Our approach is facile, one-step, aqueous-phase, and without organic solvent or heating, which produced uniform PtPdCu PNDs with a great surface area and multiple pores, which are highly beneficial for various electrocatalytic applications.

### 3.6 Methanol oxidation performance

The MOR activity and stability of thus obtained PtPdCu PNDs were compared with commercial Pt/C catalyst (20 wt % Pt, Alfa Aesar), PtPd PNDs, and PtCu PNDs. The CV curves display the voltammogram features of Pt-based catalysts comprising the three potential areas assigned to under-potential H_2ads/des_ from −0.2 to 0.13 V, double layer at 0.13–0.45 V, and Pt-redox (Pt-O/Pt-H) at higher potentials. Notably, the redox potential of PtPdCu PNDs was shifted positively (0.580 V) relative to PtPd PNDs (0.576 V), PtCu PNDs (0.570 V), and Pt/C (0.510 V), as indicated by the dashed box in (Fig. S5), due to the PNDs structure and alloying effect. This implies the late formation and weakening of Pt-oxygenated species after alloying Pt with Pd/Cu. The calculated ECSAs of porous PtPdCu PNDs, PtPd PNDs, PtCu, and commercial Pt/C are about 46.9, 56.4, 16.1, and 56.2 m^2^/g. The ECSA of self-standing PtPdCu PNDs is closer to Pt/C, inferring a great surface state and exposed facets of self-standing PtPdCu PNDs. The high ECSAs of thus formed PtPdCu PNDs are owing to the PNDs morphology, which is essential for providing abundant active catalytic sites during MOR. This could be evidenced in earlier MOR onset potential (E_Onset_) of hydroxyl group (Pt-OH) adsorption on PtPdCu PNDs (0.47 V), PdCu PNDs (0.5 V), and PtPd PNDs (0.52 V) relative to Pt/C (0.54 V), which is desired for facilitating MOR (Fig. S5) [7]. The MOR oxidation potential of PtPdCu NDs was lied between PtCu NDs and PtPd PNDs due to alloying effect of Pt with Pd and Cu, but the current density of PtPdCu PNDs was significantly higher than that of its counterparts. The MOR oxidation potential (E_Oxid_) of PtPdCu PNDs (-0.13 V) is slightly lower than that of PtPd PNDs (-0.085 V) and higher than PtCu PNDs (-0.22 V) and Pt/C (-0.23 V), however PtPdCu PNDs produce a higher current density under the same applied potential, implying the higher MOR kinetics and maximized utilization of Pt in PtPdCu NDs. The formation of self-standing Pt-based electrocatalysts with great electrochemical active surface area (ECSA) is highly desired in MOR to avoid detachment and aggregation of Pt-based nanocrystals from the support, as often noticed in the commercial Pt/C.

**Fig. 5:**
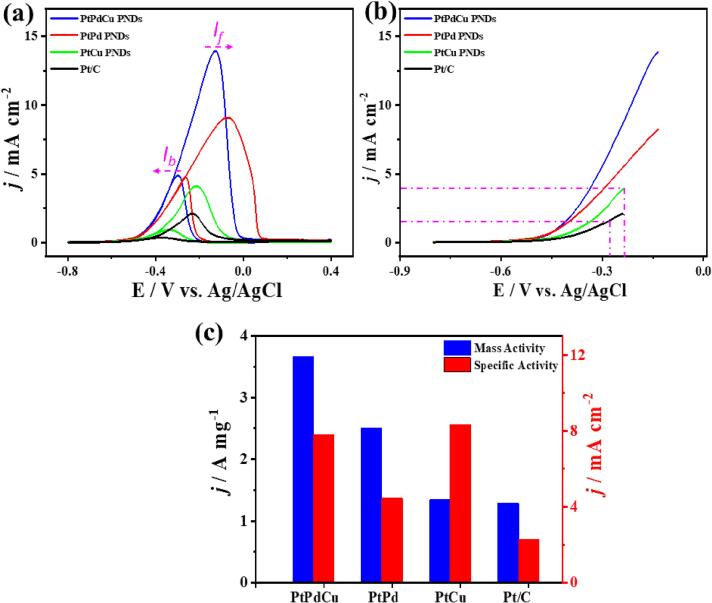
(a) CV and (b) LSV curves measured in 1.0 M KOH solution containing 1.0 M CH_3_OH at 50 mV/s and 25 °C. (c) Comparisons of the specific and mass activities.

The CV curves of thus formed PNDs and Pt/C depict the typical voltammogram characteristics of MOR comprising anodic oxidation peak current density in the forward scan (*I*_f_) for methanol oxidation and peak current in the backward scan (*I*_b_) for intermediate oxidation, but with superior activity for PtPdCu PNDs. The *I*_f_ of PtPdCu PNDs (13.9 mA/cm^2^) was greater than those of PtPd PNDs (9.1 mA/cm^2^), PtCu PNDs (4.1 mA/cm^2^), and Pt/C (2.11 mA/cm^2^), by 1.52, 3.39, and 6.58 times, respectively (Fig. 5a). This is due to the electronic effect of ternary PtPdCu PNDs containing Pd/Cu with a higher oxophilicity than Pt, which promotes the activation/dissociation of H_2_O to generate active OH* species needed for accelerating the MOR kinetics along with oxidative removal of adsorbed intermediates on Pt surface. Also, the porous structure provides confined space with promoted molecular interaction enhances the MOR activity and kinetics. This is evidenced in the lower MOR onset potential of PtPdCu PNDs (-0.40 V), PtPd PNDs (-0.38 V), and PtCu NDs (-0.40 V) than Pt/C (-0.37 V). The higher MOR kinetics is also seen in the LSV curves, which display the ability of PtPdCu PNDs to produce a greater *I*_f_ than PtPd PNDs, PtCu NDs, and Pt/C at a lower potential (lines in Fig. 5b). The superior MOR kinetics of PtPdCu PNDs is attributed to the alloying of Pt with Pd and Cu, which induces the generation of oxygenated species required for oxidative removal of poisoning intermediates on Pt surface. Also, porous branched shape with interconnected cavities [37], [38], [40], [41], [43] enhances the adsorption of methanol molecule and facilitates its diffusion to the stable inner cavities, and weakens the adsorption of intermediates, in addition to maximizing Pt/Pd/Cu atom usage during MOR, resulting in a rapid oxidation kinetic. That is seen in the higher MOR MA of PtPdCu PNDs (3.66 mA/µg_Pt_) than PtPd PNDs (2.51 mA/µg_Pt_), PtCu NDs (1.34 mA/µg_Pt_), and Pt/C (1.29 mA/µg_Pt_) based on an equivalent Pt mass (Fig. 5c). Meanwhile, the specific activity of PdPtCu NDs (7.8 mA/cm^2^), PtPd PNDs (4.45 mA/cm^2^), and PtPd NDs (8.34 mA/cm^2^) were superior to Pt/C (2.29 mA/cm^2^), respectively (Fig. 5c). Notably, the MOR MA of PtPdCu PNDs (based on Pt or PtPd or PtPdCu mass outperformed all previously reported PtPdCu nanostructures (Table S1), and was superior to other ternary Pt-based nanostructures reported elsewhere (Table S3)[27], [28], [29], [30], [31], [32], [37], [38], [39], [40], [41], [42], [43]. This is due to the PNDs shape with abundant pores and interior/exterior cavities, which maximizes adsorption and diffusion of reactants along with making ternary metals more accessible during MOR.

**Fig. 6:**
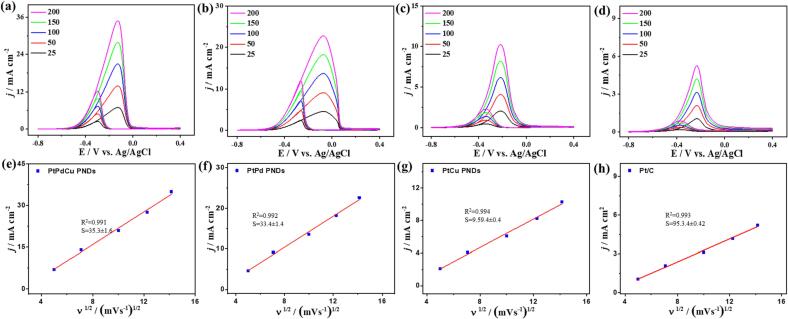
(a, b, c, d) CV curves and (e, f, g, h) their correlated plot of *I*_f_ vs. ν^1/2^ for MOR of PtPdCu PNDs (a,e), PtPd PNDs (b,f), PtCu PNDs (c,g), and Pt/C (d,h), respectively, which were measured under different scan rates in 1.0 M KOH + 1.0 M CH_3_OH electrolyte.

The LSV curves measured without methanol molecule did not exhibit any noticed current density, while in the presence of methanol, a strong oxidation current density was resolved, implying that the produced *I*_f_ is mainly from MOR (Fig. S6). The MOR was measured at different scan rates to get more insights into the electron transfer and oxidation kinetics. The *I*_f_ increases continuously with increasing the sweeping rate (ν) from 25 to 200 mV/s on all catalysts, due to the larger diffusion layer, subsequent low flux and low current are observed on the catalyst's surface at slow *ν* than at fast *ν* rate (Figs. 6a-6d). The linear relationship between *I*_f_ and *ν*^1/2^ is recorded on PNDs and Pt/C as plotted using the Randles-Sevcik equation, serving as evidence for MOR diffusion-controlled process (Fig. 6e-h) [54]. However, PtPdCu PNDs revealed a larger slope (35.3) than PtPd PNDs (33.4), PtCu PNDs (9.59), and Pt/C (5.3), inferring the quicker transportation kinetics on PtPdCu PNDs surface (Figs. 6e-6h) [54].

The MOR durability was evaluated on the as-formed PNDs catalysts relative to Pt/C using the CV curves and chronoamperometry tests in 1.0 M KOH + 1.0 M CH_3_OH at room temperature (see details in supporting information). After the durability tests, all catalysts reserved their initial MOR voltammogram features, but PtPdCu PNDs revealed the highest stability without any change in the *I*_f_ compared with its counterparts, which showed a significant degradation in the *I*_f_ as marked by the dashed boxes in (Fig. S7). Intriguingly, PtPdCu PNDs maintained 99.1 % of their initial mass activity compared with PtPd PNDs (75.0 %), PtCu PNDs (64.8 %), and Pt/C (73.8 %), due to the alloying effect of ternary metals and porous dendritic shape, which stabilize active sites against aggregation (Fig. S7a-7d). This is shown in the great stability of ECSA of PtPdCu PNDs (97.5 %) compared with those of PtPd PNDs (79.0 %), PtCu PNDs (71.0 %), and Pt/C (58.0 %) after the durability tests (Fig. S7f).

**Fig. 7:**
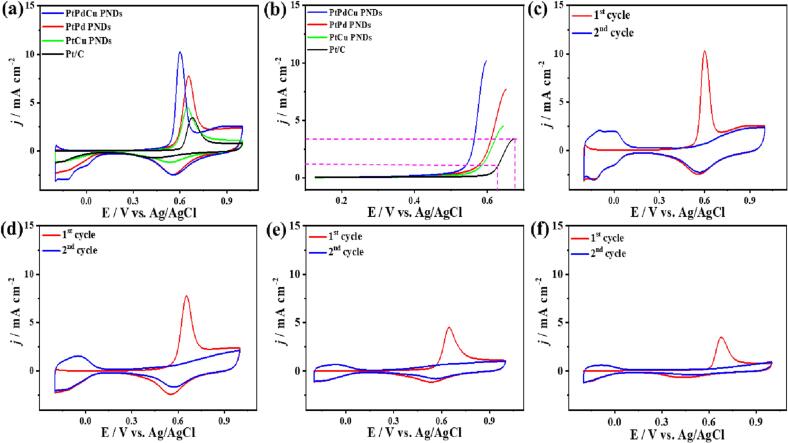
(a) CV curves of CO-stripping and (b) LSV curves for all the catalysts measured in 0.5 M H_2_SO_4_ at 50 mV/s. CO-stripping curves for (c) PtPdCu PNDs, (d) PtPd PNDs, (e) PtCu PNDs, and (f) Pt/C catalyst.

**Fig. 8:**
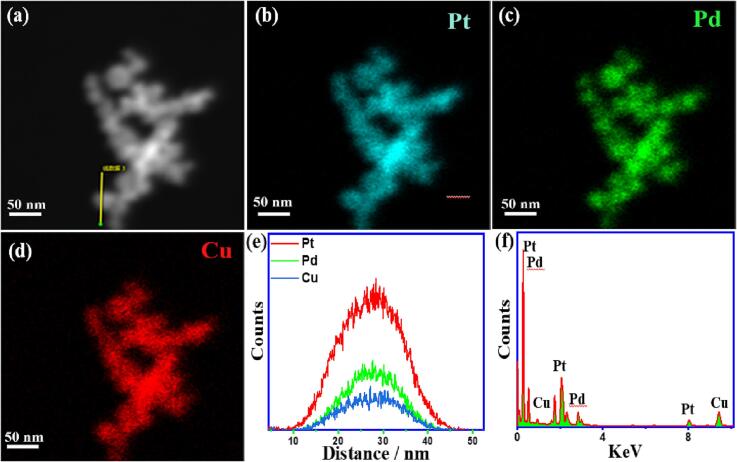
(a) HAADF-STEM image, (b-d) element mapping analyses, (e) EDS-scan line profile, and (f) EDS of PtPdCu PNDs after durability tests.

The CO-poisoning is a crucial barrier in commercializing direct methanol fuel cells (DMFCs), so the CO-stripping is measured on PNDs relative to Pt/C [53]. Under CO-pursing, all catalysts display the typical CO voltammogram features, including a clear oxidation peak in the onward direction and a minor peak in the backward direction, along with disappearing the H_2ads/des_ peak, attributed to the CO oxidation to CO_2_ (Fig. 7a). However, PtPdCu PNDs, PtPd PNDs, and PtCu revealed a lower *E*_Oxid_ and *E*_Onset_ of CO oxidation than commercial Pt/C catalyst, which implies the superior CO-tolerance of PNDs than Pt/C. The LSV curves showed the superior CO oxidation efficiency on PtPdCu PNDs than PtPd PNDs, PtCu PNDs, and Pt/C, as shown by its ability to oxidize and deliver a higher *I*_f_ value at a lower potential marked by the dashed lines in (Fig. 7b). Then, in the 2nd cycle, the CO oxidation peaks disappeared and the H_2ads/des_ in the absence of CO, which indicates the complete CO oxidation on the catalyst surface and quick recovery of adsorption sites (Fig. 7d-f) [53], [56]. The TEM image of PtPdCu PNDs after the durability tests showed the maintenance of porous dendritic shape without any morphological changes or aggregation, implying morphological durability (Fig. S8). Also, the element mapping analysis detected Pt, Pd, and Cu, and the EDS scan demonstrated the Pt-enriched shell and located Pd/Cu in the core area (Figs. 8a-8e). The EDS and element mapping revealed that the atomic contents of Pt, Pd, and Cu are about 51, 28, and 21 at.%, respectively (Figs. 8e,8f), implying compositional stability without any significant phase transition.

All results proved that ternary metal alloy and NDs endowed the MOR activity and durability of PtPdCu PNDs more than their counterparts. PNDs structure, with its interior, exterior cavities, and atomic steps, affords abundant active sites for methanol adsorption, allowing their diffusion to inner cavities, which are stable against aggregation [25], [26], [27], [28], [29], [30], [31], [32], [37], [38], [39], [40], [41], [42], [43]. That maximizes the utilization of buried metal atoms and speeds up electron mobility during MOR. To investigate the effect of sonication on the MOR activity, the CV curves were measured in N_2_-saturated 0.5 M H_2_SO_4_ at 50 mV/s on the PtPdCu PNDs prepared under sonication relative to flower-like PtPdCu prepared under magnetic stirring without sonication, both show the voltammogram features of Pt, but with a lower H_2ads/des_ area on flower-like PtPdCu than PtPdCu PNDs with sonication (Fig. S9a). The ECSA of PtPdCu flower-like without sonication (20.93 m^2^/g) was lower than that of PtPdCu PNDs with sonication (46.9 m^2^/g) (Fig. S9a). The CV curves measured on PtPdCu flower-like 1.0 M KOH + 1.0 M CH_3_OH showed a lower MOR activity and with a lower *I*_f_ (5.33 mA/cm^2^) than PtPdCu PNDs with sonication (13.9 mA/cm^2^) (Fig. S9b).

The MOR MA of PtPdCu PNDs with sonication (3.66 mA/µg_Pt_) was greater than that of PtPdCu NDs without sonication (1.34 mA/µg_Pt_). To this end, the N_2_ adsorption–desorption analysis of flower-like PtPdCu prepared without sonication reveals the isotherms feature close to the type I curve with major adsorption at 0.8 < *P/P*_o_ < 0.9 and estimated BET surface area of 22.5 m^2^∙g^−1^ (Fig. S10a). Also, flower-like PtPdCu showed less pore volume of 0.021–0.024 cm^3^/g (Fig. S10b), which implies the significantly lower BET surface area and pore volume in the flower-like PtPdCu prepared without sonication, that serves as evidence for the substantial effect of acoustic cavitation mechanism on the enhancement of porosity and surface area of PtPdCu PNDs.

The synergism of Pt-Pd-Cu increases the adsorption of methanol alongside a great CO-tolerance [25], [26], [27], [28], [29], [30], [31], [32], [37], [38], [39], [40], [41], [42], [43]. In particular, alloying Pt with Pd and Cu allows for synergistic interactions and adjustable electronic states of Pt catalyst, thus easing the adsorption of reactants along with desorption of carbonaceous intermediates (i.e., CO and HCOO), which facilitates the electron transfer and endorses splitting of C-H bond in CH_3_OH under a low potential. The presence of Pd and Cu, with their higher oxophilicity [57] than Pt promotes the H_2_O activation/dissociation in KOH electrolyte, allowing the generation of active oxygen species (i.e., O and OH*) required for facilitating and increasing the MOR activity on adjacent Pt active sites (CH_3_OH + 8OH^-^ → CO_3_^2–^ + 6H_2_O + 6e^-^), in which the MOR follows the bifunctional mechanism. This is in addition to accelerating the transformation of Pt from its oxidation state (Pt^2+^) to the metallic state Pt^o^, enabling the selective MOR path on PtPdCu PNDs. Also, Pd and Cu can provide oxygen-containing species under lower potentials than Pt, which weakens the adsorption of carbonaceous intermediates on the Pt surface and eases their oxidative removal on nearby Pt surfaces via the Langmuir-Hinshelwood (L-H) mechanism. Thereby, the MOR mechanism could be proposed as follows:

(2) $$\mathrm{Pt}+CH_{3}OH↔Pt-{\left(CH_{3}OH\right)}_{\mathrm{ads}}$$

(3) $$\mathrm{PdCu}-O+H_{2}O+2e-\to PdCu+2OH^{-}$$

(4) $$\mathrm{PdCu}+OH^{-}↔PdCu-{\left(OH^{-}\right)}_{\mathrm{ads}}$$

(5) $$\mathrm{Pt}-{\left(CH_{3}OH\right)}_{\mathrm{ads}}+5PdCu-{\left(OH^{-}\right)}_{\mathrm{ads}}\to Pt-{\left(\mathrm{HCO}O^{-}\right)}_{\mathrm{ads}}+5PdCu+4H_{2}O+4e^{-}$$

(6) $$\mathrm{Pt}-{\left(\mathrm{HCO}O^{-}\right)}_{\mathrm{ads}}+PdCu-OH_{\mathrm{ads}}\to Pt-{\left(CO_{2}\right)}_{\mathrm{ads}}+PdCu+H_{2}O+2e-$$

(7) $$\mathrm{Pt}-{\left(CO_{2}\right)}_{\mathrm{ads}}↔Pt+CO_{2}$$

## 4 Conclusion

In brief, a facile, green, and one-step approach is presented for the rational aqueous-phase design of spatial ternary PtPdCu PNDs at room temperature, driven by the sonication of a solution of Pt/Pd/Cu precursors and F127 in water, and AA as a reducing agent at 25 ℃. This is based on the isolation between nucleation and growth besides the acoustic cavitation effect of sonication. PtPdCu PNDs formed in a well-defined dendritic shape with ordered cavities-like pores distributed in the core/shell area and homogenous distribution of Pt/Pd/Cu with atomic content of 51/25/24 at.%, respectively. The MOR activity and durability of PtPdCu PNDs outperformed PtPd PNDs, PtCu PNDs, and Pt/C, along with a superior CO-poisoning tolerance, due to the tri-metals with multifunctional effect and PNDs morphology. The MOR mass activity of PtPdCu PNDs (3.66 mA/µg_Pt_) was 1.45, 2.73, and 2.83 times that PtPd PNDs, PtCu NDs, and Pt/C, respectively. Also, PtPdCu PNDs reserved their morphology and composition without significant change after the MOR durability tests.

## CRediT authorship contribution statement

**Qingqing Lu:** Conceptualization, Methodology. **Xilei Gu:** Data curation. **Jiaojiao Li:** Visualization. **Wenpeng Li:** Investigation, Supervision. **Rafael Luque:** Validation, Supervision. **Kamel Eid:** Writing – review & editing, Supervision.

## Declaration of Competing Interest

The authors declare that they have no known competing financial interests or personal relationships that could have appeared to influence the work reported in this paper.
