# Supplementary material for: Unraveling ultrasonic assisted aqueous-phase one-step synthesis of porous PtPdCu nanodendrites for methanol oxidation with a CO-poisoning tolerance
Source: Ultrason Sonochem. 2023 Jun 17;98:106494. doi: 10.1016/j.ultsonch.2023.106494 (PMC10319326; doi:10.1016/j.ultsonch.2023.106494)
Supplement: Supplementary data 1 [file mmc1.docx]

**Supporting information**

Unraveling Ultrasonic Assisted Aqueous-phase One-Step Synthesis of Porous PtPdCu Nanodendrites for Methanol Oxidation with a CO-poisoning Tolerance

Qingqing Lu,^a^ Xilei Gu,^a^ Jiaojiao Li,^a^ Wenpeng Li,^a^ Rafael Luque^b,c^ and Kamel Eid^d*^

1. Engineering & Technology Center of Electrochemistry, School of Chemistry and Chemical Engineering, Qilu University of Technology (Shandong Academy of Sciences), Jinan, China
2. Peoples Friendship University of Russia (RUDN University), 6 Miklukho Maklaya str., 117198, Moscow, Russian Federation
3. Universidad ECOTEC, Km 13.5 Samborondón, Samborondón, EC092302
4. Gas processing center (GPC), College of Engineering, Qatar University, Doha 2713, Qatar. *E-mail: kamel.eid@qu.edu.qa

*Corresponding author e-mail: kamel.eid@qu.edu.qa

**1. Methanol Oxidation Reaction**

All electrochemical tests were measured on a CHI 760E (Chenhua, China) potentiostat using a three-electrode glass cell of platinum wire (counter electrode), Ag/AgCl (saturated KCl, reference electrode), and glassy carbon ((GCE), 3 mm diameter, working electrode). The GCE was polished using alumina powder (0.3 μm and 0.05 μm) followed by washing with ethanol/deionized H_2_O (3/1 v/v) under sonication at 25 ^o^C. The cleaned GCE was coated with a 2 µg of each catalyst ink (2 mg/ml ethanol/H_2_O/Nafion 3/1/0.05 volume ratio) and then left to dry under vacuum at 50^o^C before measurements. The electrochemically active surface areas (ECSAs) were calculated using (Eq. 1)

ECSAs= Q_H_/m×210 Eq. 1

Where Q_H_ is the charge for hydrogen desorption after the double layer correction region, m is the loading amount of metal on the GCE, and 210 µC/cm^2^ is the charge required for monolayer adsorption of hydrogen onto the Pt surface. The catalyst's loading amounts on the GCE were determined by inductively coupled plasma spectroscopy (ICP-AES, Thermo Fisher Scientific, USA). The long-term MOR durability tests of the as-synthesized catalysts were conducted by the cyclic voltammogram (CVs) and chronoamperometry tests. First of all, the chronoamperometry test was conducted on each catalyst for 5000 sec in an aqueous solution of 1.0 M KOH containing 1.0 M methanol at -0.23 V, then the CVs were measured in a fresh 1.0 M KOH solution under N_2_ for 20 cycles at 200 mV/s and then three cycles at 50 mV/s to see the change in the ECSA. Finally, the CVs were measured in a fresh solution of 1.0 M KOH containing 1.0 M methanol at 50 mV/s to check the MOR durability on each catalyst.

For the CO stripping voltammetry, the electrode was held at 0.1 V in 0.5 M H_2_SO_4_ under CO-pursing for 15 min, then the potential stepped back to 0.05 V, and the solution was pursued with N2 for 20 min to eliminate any adsorbed residual CO dissolved in the solution. Finally, the CO stripping profiles were acquired by sweeping the potential from -0.2 V to 1.0 V at a sweep rate of 50 mV/s.

1. **Calculation of the lattice strain**

The lattice strain (ε) was calculated using the Williamson-Hall method (uniform deformation model) using the following equation.^[1]^

$\beta_{hkl} cos\theta_{hkl}=\frac{k}{D}+4\varepsilon sin\theta_{hkl}$ (3)

Where $\beta_{hkl}$, $\beta_{D}$, and $\beta_{\varepsilon}$ are the XRD peak broadening, crystallite size, and strain-induced broadening, respectively. Then, plotting $\beta_{hkl} cos\theta_{hkl}$ vs. $4 sin\theta_{hkl}$ to get the slope that represents the lattice strain

1. **CO-stripping**

This involves the initial electrochemical adsorption of a monolayer of CO on thus formed PNDs and Pt/C in an aqueous solution of 0.5 M H_2_SO_4_ at 0.1 V for 900 s followed by degassing the electrolyte solution by N_2_-pursuing for 20 min to ensure elimination of CO before CVs measurements.

Table S1 Comparison of the preparation method, morphology, and MOR performance of our newly developed PtPdCu PNDs with all the previous reported PtPdCu nanostructures measured under similar conditions

| Catalysts | morphology | Preparation methods | Electrolyte | MASS Activity  (A/mg_Pt_) | Ref. |
| --- | --- | --- | --- | --- | --- |
| PtPdCu | Porous nanodendrites | One-step ultrasonic irradiation of an aqueous solution of metal slats in F127 and ascorbic acid at room temperature | 1M KOH  +1M CH_3_OH | 3.66 A/mg_Pt_  2.84 A/mg_Pd+Pt_  2.31A/mg_Pt+Pd+Cu_ | This work |
| Pt_5_PdCu_5_ | Hexapods | Solvothermal reaction of trioctylphosphine oxide, Oleylamine, dimethylformamide at 200 ^o^C for 3h followed by centrifugation and washing with ethanol and cyclohexane | 0.5M H_2_SO_4_  +0.5M CH_3_OH | 0.97 | ^[2]^ |
| PtPdCu | Nanodendrites | Chemical reduction by ascorbic acid of an aqueous solution of cetyltrimethylammonium chloride (CTAC), KOH at 90 ^o^C for 2.5 h and then centrifugation washing with water and ethanol | 0.5M H_2_SO_4_  +0.5M CH_3_OH | 0.52 | ^[3]^ |
| PtPdCu | Concave nanooctahedra | The initial synthesis of PtCu seed by the reduction in polyallylamine hydrochloride and formaldehyde solution at 140 ^o^C for 4h and then the addition of Pd slat and heating to 140 ^o^C for 4h and finally centrifugation and washing with water | 0.5M H_2_SO_4_  +0.5M CH_3_OH | 0.53 | ^[4]^ |
| PtPdCu TiN NRs | Nanorods | TiN NRs were initially grown on carbon paper via seed-assisted method at 350 °C followed by a hydrothermal process at 150 °C for 15 h, annealing in air at 550 °C for 2 h, and ammonia at 900 ^o^C for 2 h. Finally, PtPdCu thin-film was sputter-deposited onto the TiN NRs and heated at 400 ^o^C under 5 % H_2_/Ar | 0.5M H_2_SO_4_  +0.5M CH_3_OH | 0.366 | ^[5]^ |
| PtPdCu | Nanoporous spheres | Chemical reduction of an aqueous solution of F127, HCl, and metal slats by ascorbic acid at 95 ^o^C for 4 h and then centrifugation washing with water and ethanol | 0.5M H_2_SO_4_  +0.5M CH_3_OH | 0.43 | ^[6]^ |
| PtPdCu | Nanodendrites | First, electrodeposition of cylindrical nanowires arrays inside the channels of aluminum anodic oxide template, followed by a wet-chemical modification. | 0.5M H_2_SO_4_  +1.0M CH_3_OH | 0.688 | ^[7]^ |
| PdPtCu | Mesoporous sphere | Chemical reduction of metal slats in HCl and F127 solution by ascorbic acid and heating at 95^o^C for 4h | 0.5M H_2_SO_4_  +0.5M CH_3_OH | 0.430 | ^[8]^ |
| PtPdCu NDs | Nanodendrites | Chemical reduction of metals in an aqueous solution of CTAC at 95 °C for 40 min | 0.1M KOH  +0.5M CH_3_OH | 1.447  mA/µg_metal_ | ^[9]^ |
| Pt_5_PdCu_5_ | Spherical~~-~~network | Hydrothermal reaction in an aqueous solution of water, ethylene glycol, and Pluronic P123 at 180^o^C for 12 h | 0.5M H_2_SO_4_  +0.5M CH_3_OH | 0.845  mA/µg_Pt+Pd_ | ^[10]^ |
| Pt_34_Pd_33_Cu_33_ | Defect-rich nanocrystals | Chemical reduction with ascorbic acid in an aqueous solution of CTAC and citric acid for 20 minutes and then autoclave at 190 °C for 4 h | 0.1M HClO_4_+0.5M CH_3_OH | 0.66 | ^[11]^ |
| PtPdCu/rGO | Thin film | Chemical reduction in oil/water interface at room temperature | 0.5M H_2_SO_4_  +0.5M CH_3_OH | 0.296 (0.7 V, NHE) | ^[12]^ |
| Pt_50_Pd_50_Cu_50_ | Ultrathin nanowires | Chemical reduction of metal salts in the presence of Triton X-114 at room temperature | 0.5M H_2_SO_4_  +1.0M CH_3_OH | 1.505  mA/µg_Pt+Pd_ | ^[13]^ |
| Pt_0.75_Pd_0.13_Cu_0.12_ | Hexameric octahedron | Seed-mediated growth in octadecylamine and cyclohexane at 110 ^o^C and then at 270 ^o^C | 0.1M HClO_4_+1.0M CH_3_OH | 3.47 mA/cm^2^ | ^[14]^ |
| PtPdCu | Hexapod concave rhombic dodecahedrons | Chemical reduction of metals salts in an aqueous solution of NaCl, oleylamine, and cetyltrimethylammonium bromide followed by autoclave at 180 ^o^C for 24h | 0.5M H_2_SO_4_  +1.0M CH_3_OH | 2.23 | ^[15]^ |
| PtPdCu | Hollow sponges | Al-foil template-based method followed by the chemical etching by HNO_3_ for 2 days | 0.1M HClO_4_+1.0M CH_3_OH | 1.34 | ^[16]^ |


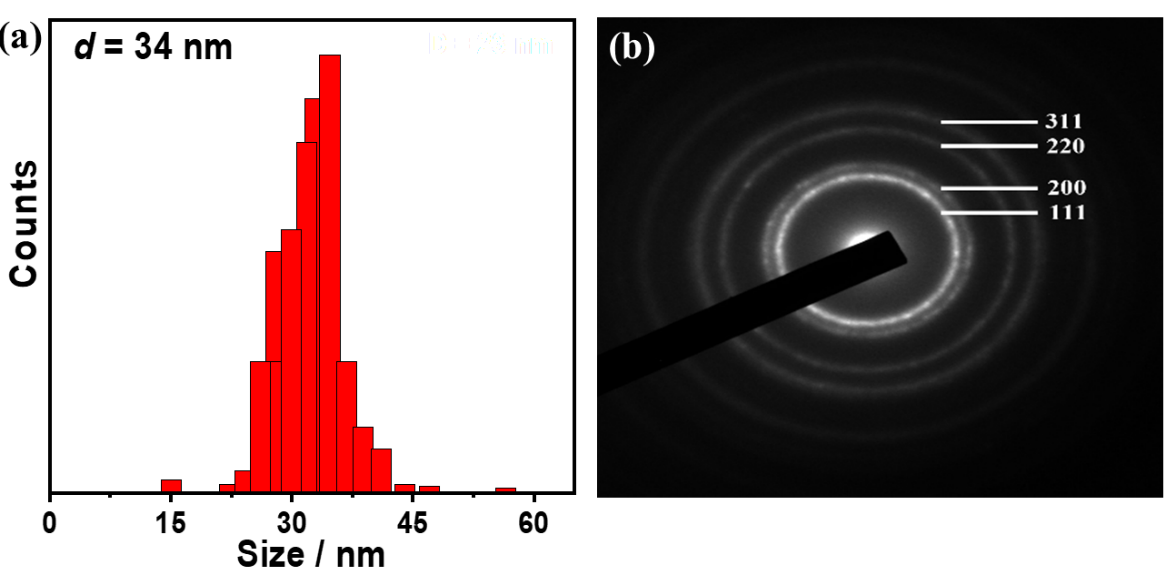


Fig. S1 (a) particle size distribution histogram and (b) SAED patterns of PtPdCu PNDs

Table S2 The composition of PtPdCu PNDs, PtPd, and PtCu PNDs determined using EDX, XPS, and ICP-OES

| Electrocatalysts | EDX  Pt/Pd/Cu | ICP-OES  Pt/Pd/Cu | XPS  Pt/Pd/Cu |
| --- | --- | --- | --- |
| PtPdCu | 51/28/21 | 52/29/19 | 53/27/20 |
| PtPd | 62/38 | 63/37 | 64/36 |
| PtCu | 57/43 | 58/42 | 55/45 |


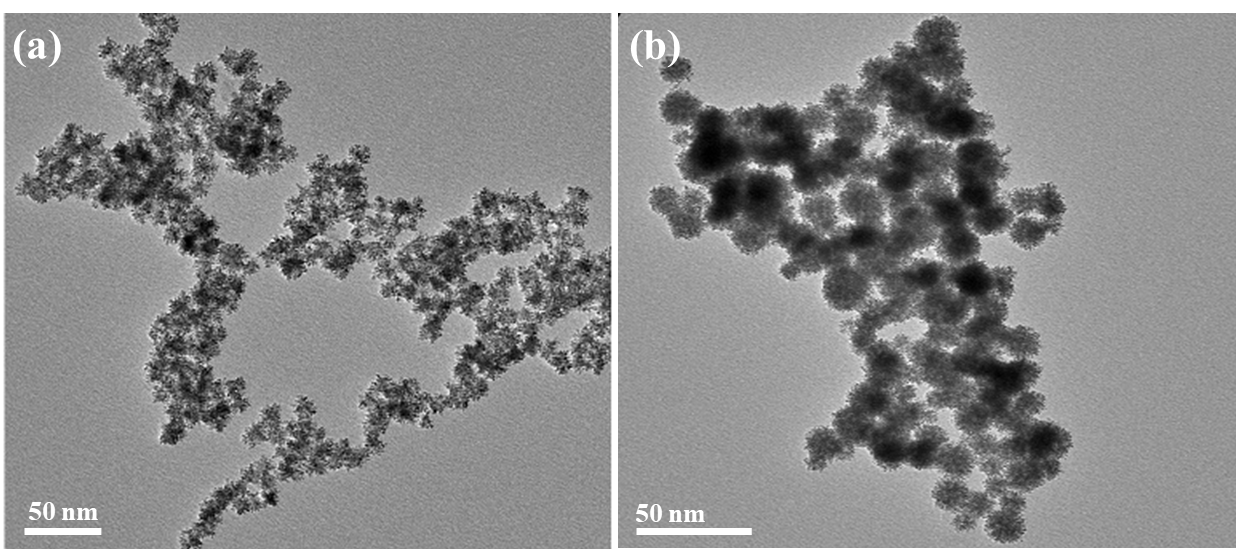


Fig. S2 TEM image of (a) PtPd PNDs and (b) PtCu PNDs

**
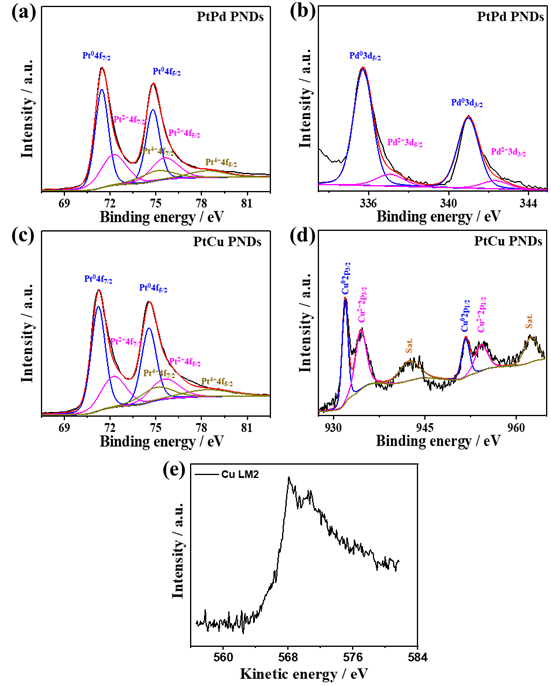
**

Fig. S3 High-resolution XPS spectra of (a) Pt4f and (b) Pd3d in PtPd PNDs. XPS spectra of (c) Pt4f and (d) Cu2p in PtCu PNDs. (e) Auger spectra of Cu LM2 in PtPdCu PNDs


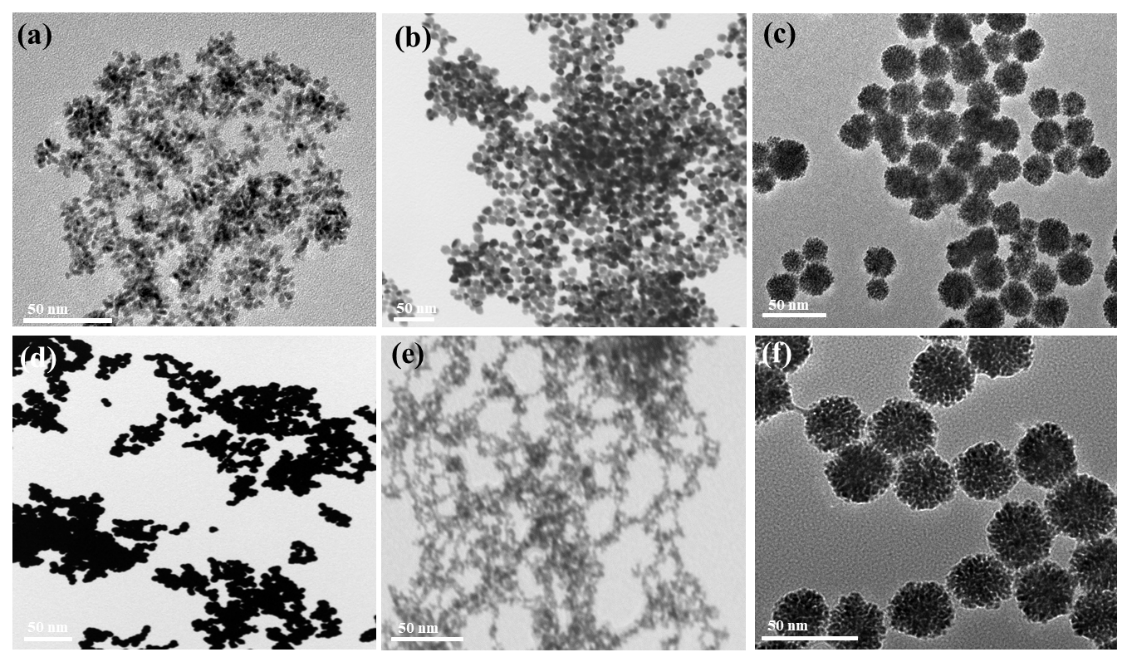


Fig. S4 TEM image of (a) Pt NDs, (b) Pd nanoparticles, (c) PtPdCu formed using PVP instead of F127, (d) PtPdCu formed without F127, (e) PtPdCu formed using HCl (0.1 M, 0.2 mL) with F127, and (f) PtPdCu formed under magnetic stirring without sonication.


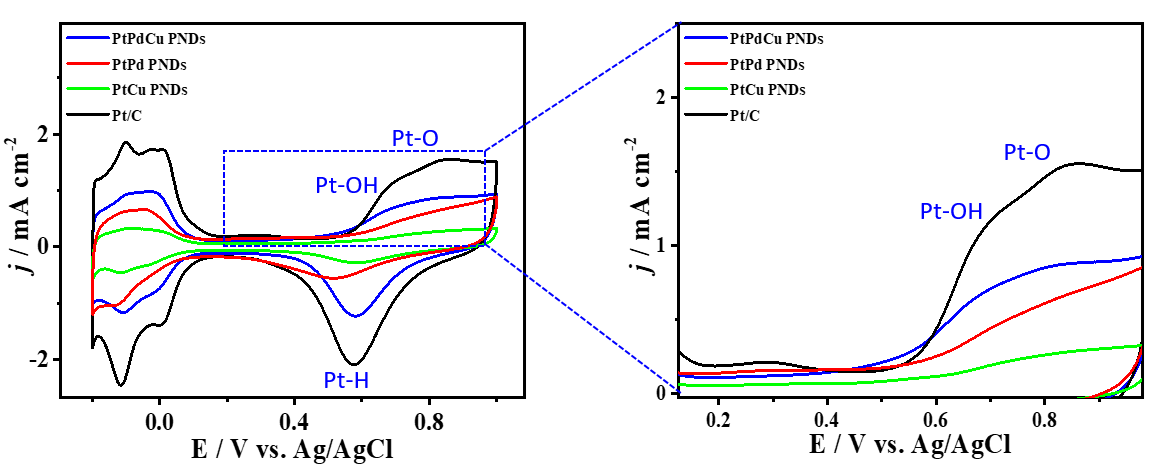


Fig. S5 CVs measured in N_2_-saturated an aqueous solution of 0.5 H_2_SO_4_ at 50 mV/s. Pt-O refers to the formation of Pt-oxide, Pt-H refers to the reduction of Pt-O


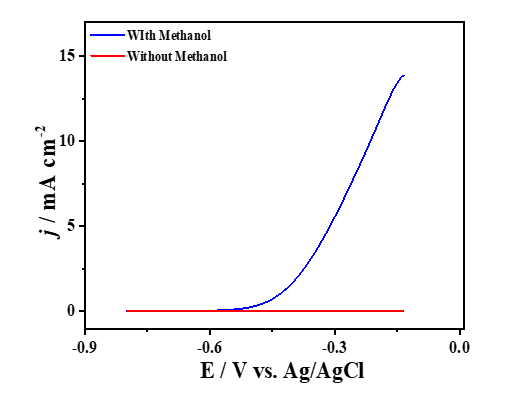


Fig. S6 LSV measured in an aqueous solution of 1.0 M KOH at 50 mV/s in the presence and absence of 1 M methanol.

Table S3 Comparison of the MOR activity of PtPdCu with elsewhere reports under the same reaction conditions

| Catalysts | Electrolyte | Mass Activity  (A/mg) | Specific Activity  (mA cm^-2^) | References |
| --- | --- | --- | --- | --- |
| PtPdCu PNDs | 1MKOH+1MCH_3_OH | 3.66 A/mg_Pt_  2.84 A/mg_Pt+Pd_  2.31 A/mg_Pt+Pd+Cu_ | 7.80 | This work |
| Pt_4_Ru_1_Cu_5_ aerogel | 1MKOH+1MCH_3_OH | 2.07 A/mg_Pt+Ru_ | 4.1 | ^[17]^ |
| Pt-Pd-Cu nanodendrites | 0.1MKOH+0.5MCH_3_OH | 1.447  A/mg_Pt+Pd+Cu_ | 3.6 | ^[9]^ |
| CuPtAu nanotubes | 1MKOH+1MCH_3_OH | 1.96 | 6 | ^[18]^ |
| Pt/Ni(OH)_2_/rGO | 1MKOH+1MCH_3_OH | 1.236 | 1.93 | ^[19]^ |
| Pt@TiO_2_/graphene | 1MKOH+1MCH_3_OH | 3.165 | 6.14 | ^[20]^ |
| Pt-Ce(CO_3_)OH/rGO | 1MKOH+1MCH_3_OH | 1.4775 | 2.45 | ^[21]^ |
| Pd_59_Cu_33_Ru_8_ NSs | 1MKOH+1MCH_3_OH | 1.6608  A/mg_Pd_ | 4.7 | ^[22]^ |
| Pt/g-C_3_N_4_/MoS_2_ | 1MKOH+1MCH_3_OH | 1.618 |  | ^[23]^ |
| Mesoporous PtRh nanosheets | 1MKOH+1MCH_3_OH | 0.521 | 0.73 | ^[24]^ |
| PtCu nanoframes | 0.5MKOH+1MCH_3_OH | 2.26 | 18.2 | ^[25]^ |


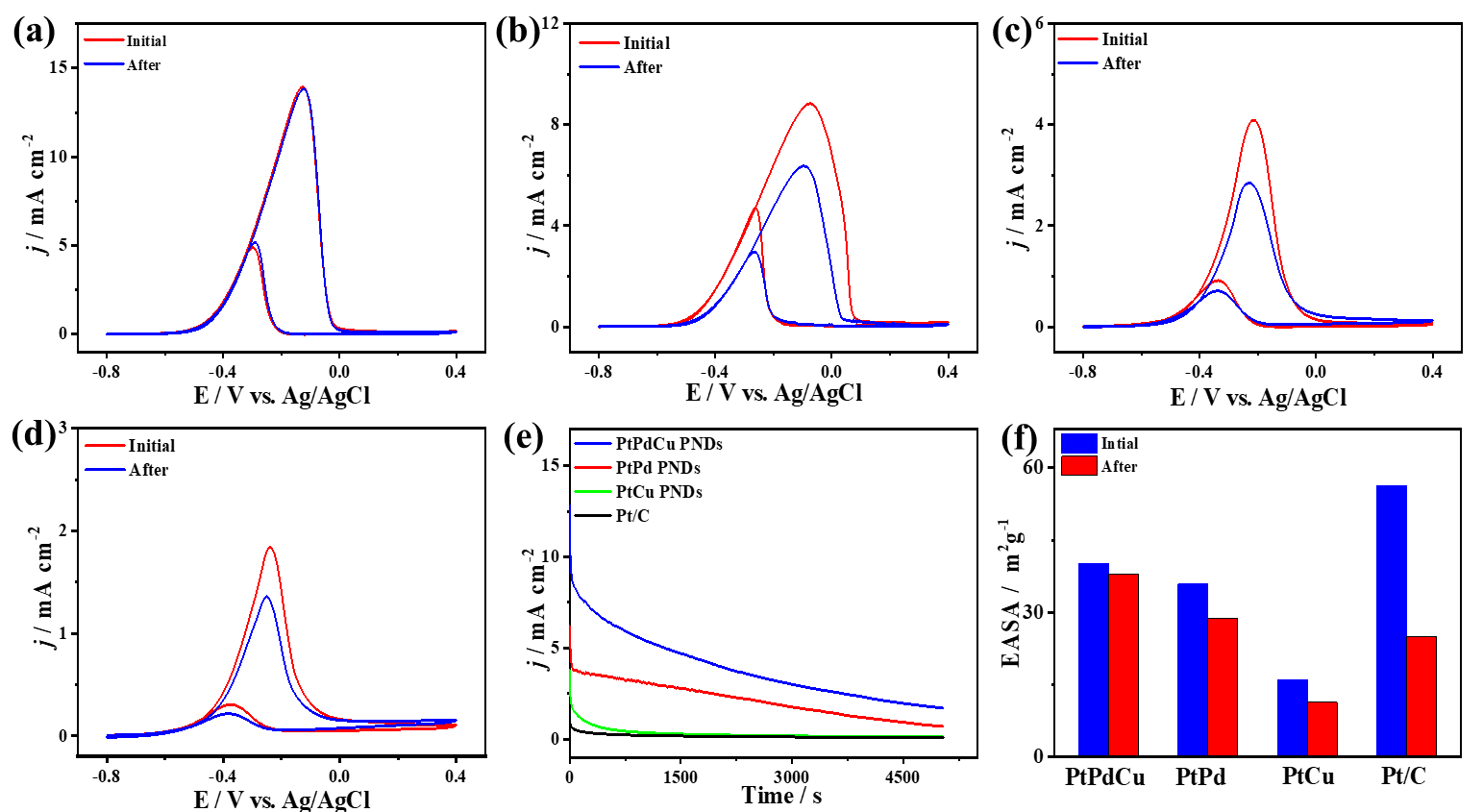


Fig. S7 MOR durability tests of (a) PtPdCu PNDs, (b) PtPd PNDs, (c) PtCu PNDs, and (d) Pt/C. (e) chronoamperometry tests measured in an aqueous solution of 1.0 M KOH with 1.0 M methanol at -0.23 V and (f) Comparison of the ECSA before and after durability tests.


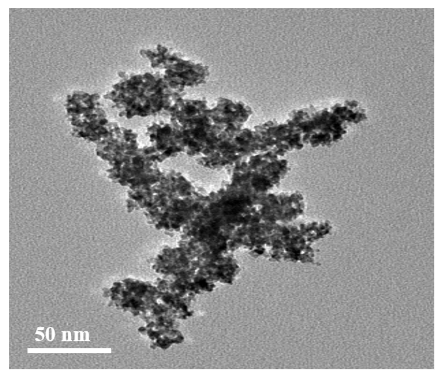


Fig. S8 TEM image of PtPdCu PNDs after durability tests


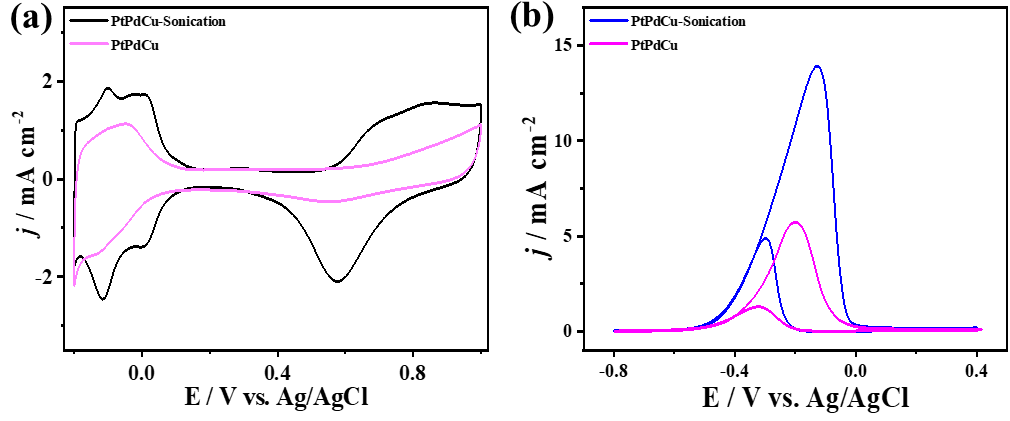


Fig. S9 (a) CV without and (b) CV with 1M methanol tested in an aqueous solution of 1.0 M KOH at 50 mV/s.


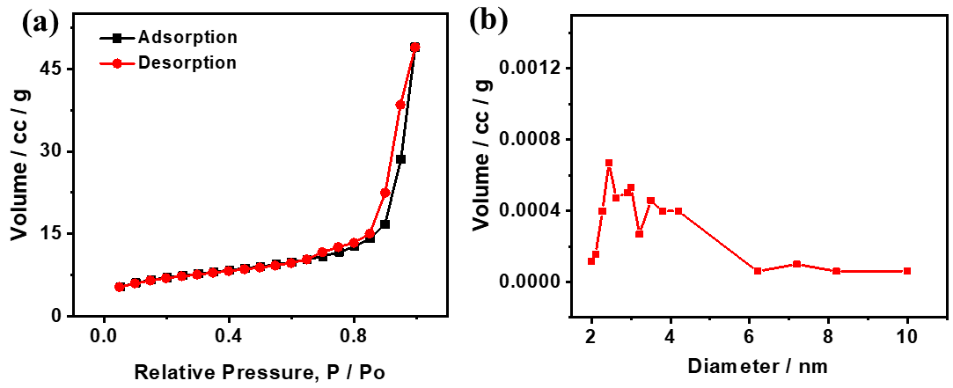


Fig. S10. (a) N_2_-adsorption/desorption isotherm and (b) pore volume of PtPdCu NDs prepared without sonication

**References**

[1] C. Suryanarayana, N. Grant, New York (1998).

[2] N. Gao, X. Wu, X. Li, J. Huang, D. Li, D. Yang, H. Zhang, RSC Adv.10 (2020) 12689-12694.

[3] P. Wang, Y. Zhang, R. Shi, Z. Wang, ACS Appl Energy Mater. 2 (2019) 2515-2523.

[4] M.-X. Gong, X. Jiang, T.-Y. Xue, T.-Y. Shen, L. Xu, D.-M. Sun, Y.-W. Tang, Catal Sci Technol. 5 (2015) 5105-5109.

[5] S. Jiang, B. Yi, Q. Zhao, H. Zhang, Y. Su, H. Yu, Z. Shao, RSC Adv. 6 (2016) 82370-82375.

[6] B. Jiang, C. Li, V. Malgras, Y. Yamauchi, J Matter Chem A 3 (2015) 18053-18058.

[7] R. Chang, L. Zheng, C. Wang, D. Yang, G. Zhang, S. Sun, Appl Catal B Environ. 211 (2017) 205-211.

[8] B. Jiang, C. Li, M. Imura, J. Tang, Y. Yamauchi, Adv Sci. 2 (2015) 1500112.

[9] Y.W. Lee, M. Im, J.W. Hong, S.W. Han, ACS Appl Mater Interfaces 9 (2017) 44018-44026.

[10] Y. Fan, Y. Zhang, Y. Cui, J. Wang, M. Wei, X. Zhang, W. Li, RSC Adv. 6 (2016) 83373-83379.

[11] J. Lan, K. Wang, Q. Yuan, X. Wang, Mater Chem Front. 1 (2017) 1217-1222.

[12] S.J. Hoseini, M. Bahrami, Z. Samadi Fard, S. Fatemeh Hashemi Fard, M. Roushani, B.H. Agahi, R. Hashemi Fath, S.S. Sarmoor, Int J Hydrogen Energy 43 (2018) 15095-15111.

[13] X. Zhao, J. Zhang, L. Wang, H.X. Li, Z. Liu, W. Chen, ACS Appl Mater Interfaces 7 (2015) 26333-26339.

[14] J. Mao, T. Cao, Y. Chen, Y. Wu, C. Chen, Q. Peng, D. Wang, Y. Li, Chem Commun. 51 (2015) 15406-15409.

[15] H. Chen, R. Wu, P.K. Shen, ACS Sustain Chem Eng. 8 (2020) 1520-1526.

[16] Y.-X. Xiao, J. Ying, G. Tian, X. Yang, Y.-X. Zhang, J.-B. Chen, Y. Wang, M.D. Symes, K.I. Ozoemena, J. Wu, X.-Y. Yang, Nano Lett. 21 (2021) 7870-7878.

[17] H. Wang, Y. Wu, X. Luo, L. Jiao, X. Wei, W. Gu, D. Du, Y. Lin, C. Zhu, Nanoscale 11 (2019) 10575-10580.

[18] N. Naresh, P. Karthik, R. Vinoth, C. Muthamizhchelvan, B. Neppolian, Electrochimica Acta 282 (2018) 792-798.

[19] W. Huang, H. Wang, J. Zhou, J. Wang, P.N. Duchesne, D. Muir, P. Zhang, N. Han, F. Zhao, M. Zeng, Nat commun. 6 (2015) 1-8.

[20] K. Zhang, J. Qiu, J. Wu, Y. Deng, Y. Wu, L. Yan, J Matter Chem A (2022).

[21] G. Chen, Z. Dai, L. Sun, L. Zhang, S. Liu, H. Bao, J. Bi, S. Yang, F. Ma, J Matter Chem A 7 (2019) 6562-6571.

[22] L. Jin, H. Xu, C. Chen, H. Shang, Y. Wang, C. Wang, Y. Du, ACS appl mater Interfaces 11 (2019) 42123-42130.

[23] C. Zhai, M. Sun, L. Zeng, M. Xue, J. Pan, Y. Du, M. Zhu, Appl Catal B Environ. 243 (2019) 283-293.

[24] S. Wang, Q. Mao, H. Ren, W. Wang, Z. Wang, Y. Xu, X. Li, L. Wang, H. Wang, ACS Nano (2022).

[25] Z. Zhang, Z. Luo, B. Chen, C. Wei, J. Zhao, J. Chen, X. Zhang, Z. Lai, Z. Fan, C. Tan, Adv Mater. 28 (2016) 8712-8717.
